# On-Barn Pig Weight Estimation Based on Body Measurements by Structure-from-Motion (SfM)

**DOI:** 10.3390/s18113603

**Published:** 2018-10-24

**Authors:** Andrea Pezzuolo, Veronica Milani, DeHai Zhu, Hao Guo, Stefano Guercini, Francesco Marinello

**Affiliations:** 1 Department of Agroforesty and Landscape, University of Padua, 35020 Legnaro, Italy; veronica.milani@studenti.unipd.it (V.M.); stefano.guercini@unipd.it (S.G.); francesco.marinello@unipd.it (F.M.); 2 College of Land Science and Technology, China Agricultural University, Beijing 100083, China; zhudehai@cau.edu.cn (D.Z.); guohaolys@cau.edu.cn (H.G.)

**Keywords:** pig barn, automatic measurement, animal weight, three-dimensional reconstruction, structure from motion

## Abstract

Information on the body shape of pigs is a key indicator to monitor their performance and health and to control or predict their market weight. Manual measurements are among the most common ways to obtain an indication of animal growth. However, this approach is laborious and difficult, and it may be stressful for both the pigs and the stockman. The present paper proposes the implementation of a Structure from Motion (SfM) photogrammetry approach as a new tool for on-barn animal reconstruction applications. This is possible also to new software tools allowing automatic estimation of camera parameters during the reconstruction process even without a preliminary calibration phase. An analysis on pig body 3D SfM characterization is here proposed, carried out under different conditions in terms of number of camera poses and animal movements. The work takes advantage of the total reconstructed surface as reference index to quantify the quality of the achieved 3D reconstruction, showing how as much as 80% of the total animal area can be characterized.

## 1. Introduction

The live weight (LW) of pigs is an important factor for managing various stages of its supply-chain [1]. At the farm scale, LW data is of great interest for pig management, as it serves as an index for estimate animal growth [2], feed conversion efficiency [3], and health and disease occurrence [4,5]. Moreover, the ability to accurately estimate pig sizes before setting up harvesting [6] or procurement plans is very important for the stockman and the industry [7].

Currently, most assessments of LW or pig conformation are conducted by eye and hand, based on the (subjective) experience of the observer [8] and/or by direct weighing of the animal [9]. Although, direct weighing method provides the most accurate output, it involves a cumbersome and time-consuming task [10] and could cause injury and stress to animals and stockmen when forcing the animal onto the scale. Girth measurement is one of the main indirect approaches and also applied in pig weighing [11]. However, this approach is laborious and difficult, and it may be stressful for both the animals and the stockman [12]. 

Alternatively, optical sensing systems can be implemented in order to overcome difficulties arising from direct measurements [13,14]. Animals are typically imaged from the top and only seldom imaged from the side by CCD (charge-coupled device) camera. Successively, images are processed by specialized software in order to correlate the extracted features (e.g., projected area or profile) to the pig’s LW.

Different kinds of optical sensor have been used, as for instance 2D cameras, but there is a greater interest for 3D sensors, such as TOF (time of flight) cameras or CTS (consumer triangulation sensor) systems [15]. Furthermore, approaches for pig LW estimation by means of a Microsoft Kinect^®^ camera have been proposed, taking advantage of infrared depth map images [16,17,18]. In particular, Kongsro [19] reported weight estimation results from Landrace and Duroc breeds in the 20–140 kg body weight interval, based on volume measurements. Recently, a number of different hardware-software solutions have been also proposed in the market, to estimate a marked pig’s weight individually, such as Weight-Detect (PLF-Agritech Europe, Edinburgh, UK), Pigwei (Ymaging, Barcelona, Spain), eYeScan (Fancom BV, Panningen, The Netherlands), Growth Sensor (GroStat, Newport, UK), OptiSCAN (Hölscher, Emsburen, Germany), and WUGGL One (WUGGL, Lebring, Österreich) [20]. Most of these are either based on three-dimensional images taken by either two- or three-dimensional cameras [12,21]; however, they typically operate without clear information about data analysis procedures or uncertainties. 

Many authors have investigated on three-dimensional reconstruction methods based on optical systems, however, Structure from Motion (SfM) has been attracting attention as one of the most practical approaches for 3D reconstruction. Based on photogrammetry, SfM is a range imaging technique that estimates 3D structuring from a series of 2D images captured at different points of view all around the object [22]. SfM differs fundamentally from 2D imaging techniques in that the geometry of the scene, camera positions, and orientation are solved automatically without the need to specify targets with known 3D positions [14]. These are solved simultaneously using a highly redundant iterative bundle adjustment procedure based on a database of features automatically extracted from a set of multiple overlapping images [23].

Several studies have been proposed implementing such technique in order to collect 3D data about plant’s phenology [24], soil topography and roughness [25,26] and building reconstruction [27,28], however, SfM technique could be also implemented for livestock applications. In this field, SfM can be a new convenient tool for on-barn animal reconstruction applications, also considered the fact that camera intrinsic parameters are automatically estimated during the reconstruction process and no prior calibration is needed. The potential for animal body parameters retrieval based on SfM photogrammetry approach has not been widely explored: the present paper proposes an analysis on pig body 3D reconstruction, under different conditions in terms of number of camera poses and animal movements. Data collected were eventually compared with the results from manual LW measurements, highlighting its potential and limits.

## 2. Materials and Methods

### 2.1. Structure from Motion (SfM)

**Figure 1:**
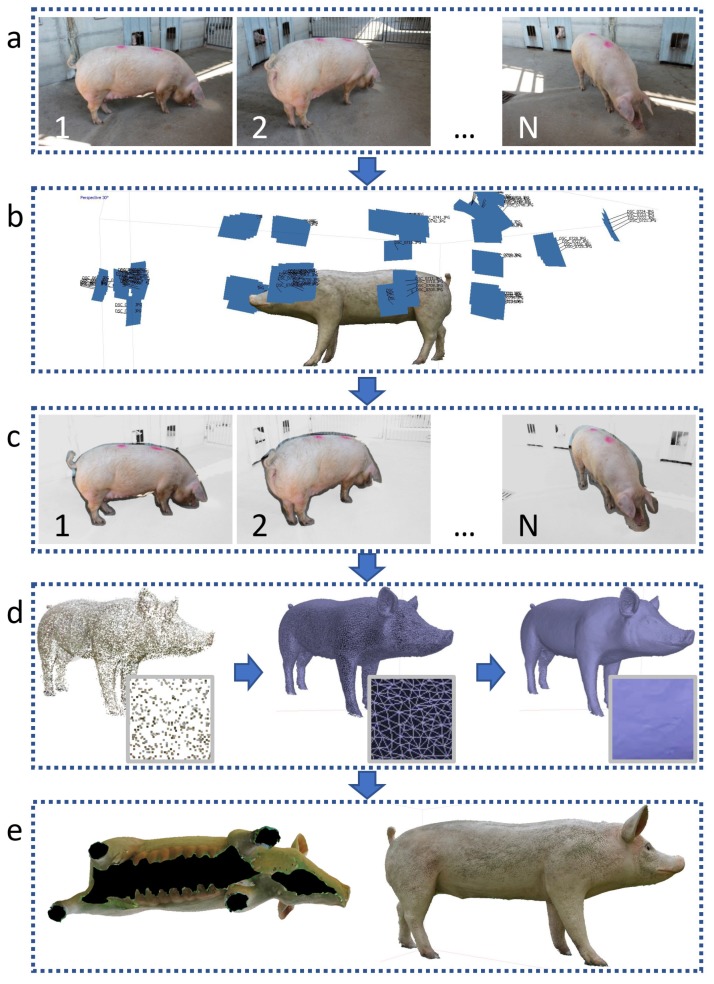
Flow chart representation of the applied methodology and 3D reconstruction procedure.

SfM is a technique which provides the three-dimensional reconstruction of any regular or free-form shape using a set of 2D images. This is implemented in some commercial software, which supports the user, thereby simplifying the need for image processing efforts. Specifically, for the present study, pigs’ reconstruction in a SfM approach was carried out through AgiSoft PhotoScan (version 1.3.1, AgiSoft LLC, St Petersburg, Russia) according to the procedure depicted in Figure 1. 

As a first step images are captured from different perspectives, homogeneously and randomly distributed all around the volume of interest (Figure 1a). Images can be collected by a single sensor moved around (as done for the present experiments), or by a set of synchronized sensors installed on a reference frame (which could be the configuration of a commercial optimized solution). The camera perspective can have an effect on the quality of 3D reconstruction, therefore in case of automation it is important to study and optimize the collocation of the positions where poses are collected. Images are initially analysed in order to recover the relative localization and the orientation of the camera from which the same images were taken, defining a set of reference positions in a Cartesian coordinate system (Figure 1b). Then the applied software implements algorithms for images registration, thus allowing automatic detection, extraction and matching of corresponding common features in multiple overlapping images (Figure 1c) and generation of a network of tie points. Such a network defines a stating reference point cloud, which constitutes the basis for subsequent refinement and definition of a dense point cloud (Figure 1d). A surface which describes the volume of interest is thus achieved: such shape undergoes a final processing which is needed in order to remove external elements (e.g., floor, background elements). Additionally, if needed, an interpolation is carried out on shape portions with missing data, such as those related to hidden or difficultly accessible parts (Figure 1e). The final shape can be also integrated with a texture in order to render a more intuitive appearance. 

The final closed dense point cloud can be eventually used to extract relevant parameters [24,25,26]. Two parameters were then selected to evaluate the quality of the reconstructed dense cloud: the surface roughness (which quantifies surface noise) and the amount of reconstructed area.

**Figure 2:**
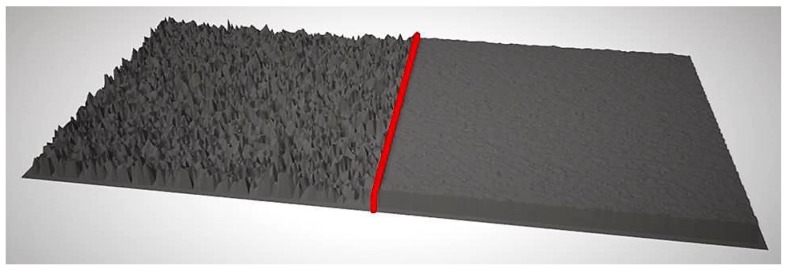
An example of a reconstructed 3D area, without (**left**) and with (**right**) application of a Gaussian filter: the applied filter reduces positive and negative peaks producing a more homogeneous and less noisy surface.

It should be pointed out how reconstructed surfaces suffer from noise, not only arising from skin roughness and outer hair, but also caused by secondary movements (due for instance to breathing, or muscle movements) which are misinterpreted during the 3D reconstruction causing a local unevenness of the texture, as can be noticed in Figure 2. 

Such noise was quantified in terms of root mean square (RMS) roughness obtaining the first parameter indicative of reconstruction quality. Since noise might apparently increase total reconstructed area, the obtained surfaces were filtered through a Gaussian filtering software tool (40 mm cut-off) in order to reduce its effects. Finally, the total reconstructed body surface was computed as a second parameter directly related to the quality of the 3D reconstruction. Area is here reported as a percentage, calculated on the basis of the expected total surface estimated from the animal weight. In order to minimize errors on total area estimation, resampled areas were excluded from computation. 

### 2.2. Animal Imaging

A commercial camera was implemented for collection of images needed for pigs’ SfM reconstruction. Specifically, a Nikon D5100 camera (Nikon Corporation, Tokyo, Japan) was used, featuring a 23.6 × 15.6 mm CMOS sensor with a 4928 × 3264 pixel resolution and a lens with a 35 mm focal length. Poses were collected considering a distance of the animals from the camera ranging between 0.7 and 1.5 m (Figure 1b). A total of at least 50 images per pig was considered, captured from withers to buttock and from the top of the back to ground. 

During the experiments, a video sequence of the scene was additionally recorded by means of a second commercial camera (GoPro Hero 4 action camera by GoPro Inc., San Mateo, CA, USA) with 8.3 MP (3840 × 2160) resolution at 30 frames per second. Such camera was positioned above the pig, in order to monitor and quantify the number of movements of the imaged animal body. The video sequence of the scene was then processed by means of a tracking software (Tracker version 4.80, Open Source Physics, California, CA, USA) [29] to digitize movements, eventually allowing estimation of frequency and amplitude of shifts from the initial position. 

### 2.3. Animals and Housing

All observations were conducted in a commercial pig weaning farm located in the North of Italy. The pigs breed was a crossbreed of Large White (50%) and Landrace (50%), and were fed with a daily ration of 2.8 ± 1.0 kg of commercial fodder mixture; water was supplied ad libitum through bite nipple drinkers.

In the pig farm, each pen barn had an average size of 8.5 m wide × 3.50 m long, divided into two areas (inside area 4.0 m wide × 3.5 m long with grid floor; outside area 4.5 m wide × 3.5 m long with solid floor), and containing 10 pigs. Pens were separated from each other by solid walls to avoid physical contact.

The temperature was automatically kept at approximately 25 °C (the temperature was adjusted if the pigs showed adverse behavioural responses), and the lighting was on a 12/12 h light/dark cycle with an average light intensity ranging between 100 and 130 lx measured at ground level using a luminance metre (Konica Minolta T10, Inc., Osaka, Japan). 

According to EU legislation (Council Directive 86/609/EEC), no procedures requiring approval from the local ethics committee were used.

### 2.4. Experimental Design and Data Acquisition

Experiments and subsequent analyses had the scope to give indications on the applicability of the SfM approach to 3D body reconstruction. To this end, analyses focused on: (i) determination of the minimum number of images needed to properly characterize body shape, (ii) quantification of the effect of animal movements on 3D reconstruction and (iii) identification of the animal body portions more likely reconstructed after application of SfM.

The first part of the research consisted of a preliminary validation of the SfM approach. For the scope, an ideal condition was created through implementation of a fiberglass reference model (Figure 1e) resembling the actual shape, posture, colour and dimensions of a real pig (Table 1). Increasing numbers of images (respectively, 10, 20, 30 40, 50, 60, 70, and 80) were captured, with a frequency of about two frames per second. Images from different perspectives and distances homogeneously distributed around the animal body were then implemented for the reconstruction of a three-dimensional model. The analysis helps to define the minimum number of frames which are needed in ideal conditions in a reconstruction process.

The fixed position and the time stability (thermal distortions of the overall length were estimated to be lower than 0.1 mm/°C) allowed also to tune the imaging and reconstruction procedure, and to calibrate the final 3D model taking advantage of the so called substitution approach [30]. Such calibration procedure is important in order to allow extraction of quantitative data from the model and, secondly. 

In the second part of the experimental campaign, 6 pigs were randomly picked from six pens. Sampled animals with an average age of 1467 ± 117 day underwent weight analyses: LW measurements were carried out by the stockman by means of an electronic scale (Laumas Electronics mod. Bil, accuracy ±0.05 kg) and a LW of approximately 258 ± 13.0 kg (mean ± s.d.) was measured (Table 1). 

Pigs were studied at the same time of the day (between 02:30 and 04:30 PM) while the animals were eating. According also to other researchers, the eating/drinking position was considered as the ideal position for the 3D reconstruction [31,32,33]; indeed, during feeding, pigs often remain in a relatively stable position for a few minutes several times a day. 

Animals included in the study exhibited different aptitudes during feeding and, thus, during experiments, mostly appearing as different amounts of movements per second. Such variability was usefully studied in order to understand the effects of body movements on 3D reconstruction. Thus, collected motion data were compared with the percentage of reconstructed body area. It is worth noting that, due to the thresholding and processing algorithms implemented into the tracking software [29], the average movements of the whole pig body were analysed through its centre of mass. Specifically, shifts of the centre position were monitored and used as an indicator of animal steadiness. Since most of pigs’ movements during feeding are typically localized in correspondence of the animal head, shifts averaged on the whole body could be somehow attenuated and, thus, underestimated. However, such kind of behaviour during feeding was systematic among different pigs, therefore the revealed parameter can still be used in a comparative way, in order to characterize the different experimental conditions. 

As already mentioned, both the number of frames and the number of movements influence the total reconstructed area. On the other hand, 3D reconstruction is more frequently and thoroughly achievable in correspondence of some body portions exhibiting a better visual accessibility which makes them to appear in a higher number of frames. Then, the second experiment was also useful in order to identify the parts of the animal body, which are more likely reconstructed after application of SfM. 

Each experiment and each animal reconstruction was repeated three times in order to have an information on the variability of the processes. 

## 3. Results and Discussion

### 3.1. Method Performances

As in the case of some other proposed techniques [10,11,12], the discussed SfM can benefit from relatively low-cost technologies (100–1000 EUR), both for the cameras and for the three-dimensional reconstruction software. Compared with classical 2D imaging [10], or with 3D Kinect imaging [12], SfM is a viable solution as highlighted also by a recent work from the same authors and here briefly summarized in Table 2 [34,35]. It can be noticed how SfM is still limited by relatively long processing time (needed to produce the 3D model using just 2D images), however, such performance is constantly increasing thanks to the availability of computers with faster and better performing processors. 

### 3.2. Influence of the Number of Frames

As reported by other researchers [24,28,36] the reconstructed surface rate is influenced by the number of frames provided to the SfM algorithms. 

**Figure 3:**
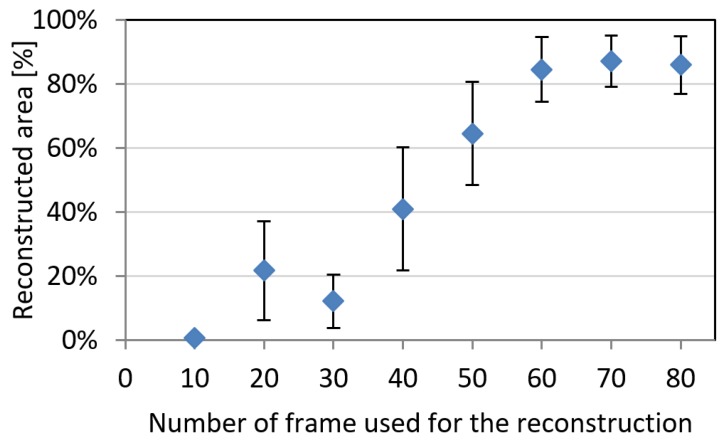
Reconstruction area with a number of frames ranging between 10 and 80. Error bars indicate standard deviation on three repeated reconstructed processes.

Experiments here proposed on the ideal fiberglass reference are in agreement with such findings: specifically, a minimum of 50 photographs is in general needed in order to guarantee the reconstruction on at least 60% of animal body (Figure 3). Such percentage excludes the bottom section comprised between the four legs, and the bottom part of the head (more difficultly imaged by surrounding photographs), which however play only a secondary role on extraction of body related parameters [12]. Then 50 frames was set as the minimum number for subsequent reconstructions on live animals. 

### 3.3. Influence of Animal’s Movements

Live animal movements are the main reason for failures or distortions in SfM 3D reconstruction. As explained above, the pig movements were quantified in terms of amplitude and frequency, highlighting clear tendencies in both of cases. 

**Figure 4:**
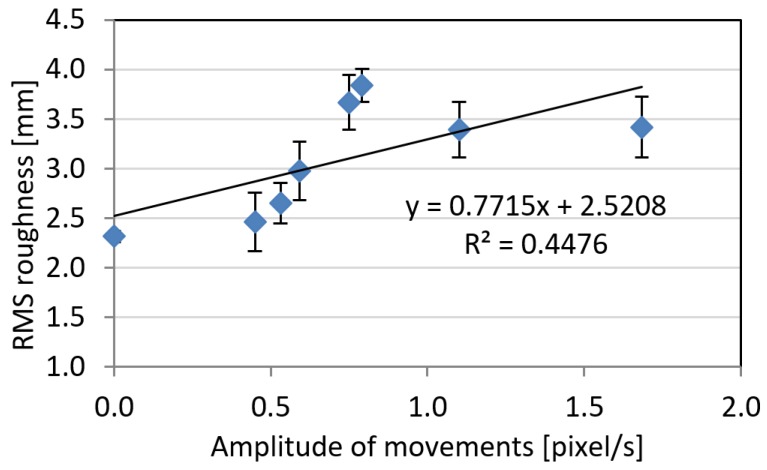
RMS roughness as a function of amplitude of movements. Error bars represent standard deviation on three different reconstructions.

**Figure 5:**
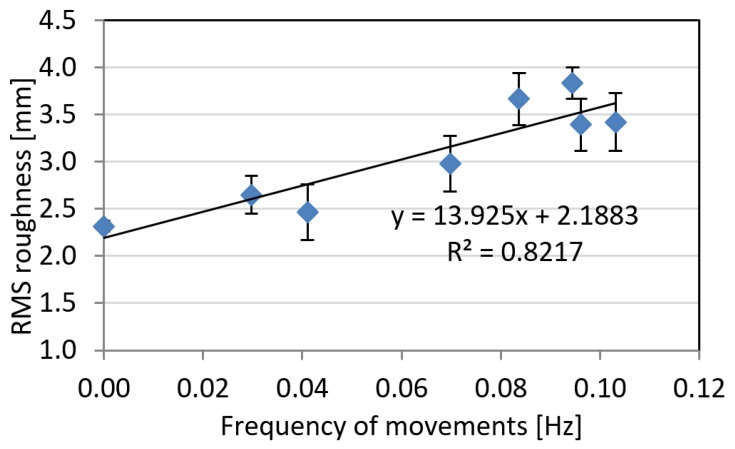
RMS roughness as a function of frequency of movements. Error bars represent standard deviation on three different reconstructions.

With regard to the residual root mean square (RMS) roughness, higher amplitude of movements in general produce higher levels of noise. The trend is characterized by a coefficient of determination R² = 0.45, which somehow indicates a certain degree of correlation (Figure 4). However, for two of the animals, intermediate values on the average amplitude of the movements caused relatively high noise levels on reconstructed surfaces. A clear trend was on the other hand highlighted by the frequency of the movements (Figure 5), which correlates with the RMS roughness with a coefficient of determination R² = 0.82. This can be explained considering the fact that animal photographs were captured at a constant frame rate: therefore, an increase on the frequency of the movements produce a corresponding increment on the total amount of animal displacements occurring during image shooting. 

**Figure 6:**
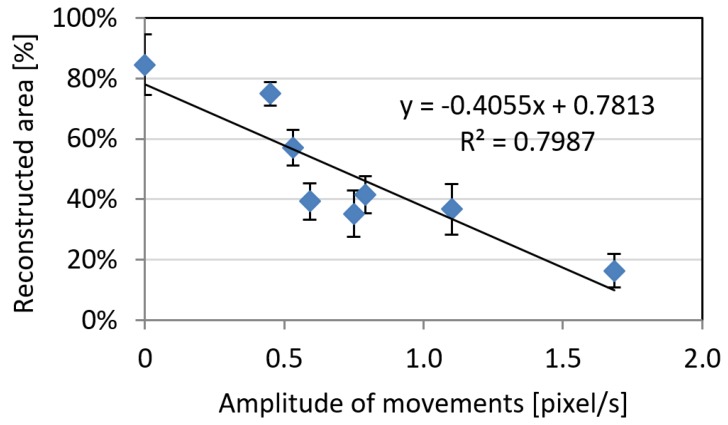
The reconstructed area rate as a function of average estimated animal movements’ amplitude. Error bars represent standard deviation on three different reconstructions.

**Figure 7:**
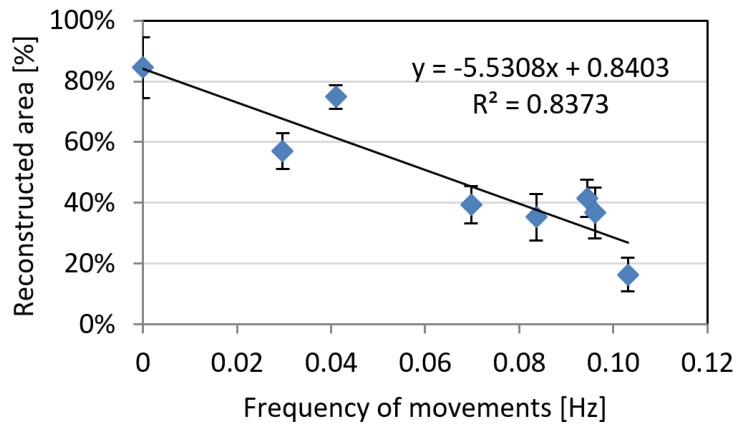
The reconstructed area rate as a function of average estimated animal movements’ frequency. Error bars represent standard deviation on three different reconstructions.

Similar trends were recognized in relation to the total amount of reconstructed areas, were lower levels of movements (both in terms of amplitude and frequency) are a condition for a good reconstruction process (Figure 6 and Figure 7). The trend is characterized by coefficients of determination R² > 0.79. It can be noticed how the behaviour for the amplitude of movements is slightly different between noise and reconstructed area: indeed, the two parameters are slightly independent and noise reconstruction can be achieved both on a highly or poorly reconstructed areas. 

Such results give clear indications of the animal scanning phase. In fact, an increase of the image acquisition frequency corresponds to an apparent reduction of the frequency and amplitude of movements entering the collected frames, and a consequent increase on the reconstruction quality. According to the results of this study, increasing the scanning speed from two to at least eight frames per seconds could guarantee a total reconstructed area larger than 60% also in the case of most nervous pigs. 

**Figure 8:**
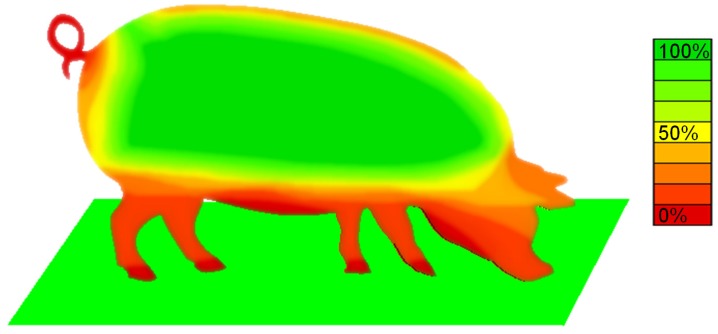
Percentage of 3D SfM processes which converged to an acceptable number of points, suitable for surface characterization.

Reconstructions on live animals were useful also in order to characterize the parts the most frequently are reconstructed after application of SfM process. A map is proposed in Figure 8, which represents the percentage of 3D body reconstructions which provided points for acceptable surface characterization. The map clearly shows how wider flat areas localized on the flanks and on the top and back of the animal are those most frequently reconstructed. This result indicates that on such animal portion, extracting body related parameters should be more easy or reliable after SfM point cloud extraction. These animal portion is clearly limited but can be enlarged in case of higher frequency image acquisition rates and is, however, interesting for calculation of parameters, such as hearth girth and animal length, which according to previous studies [11,37,38] can be linked to body condition and weight. 

## 4. Conclusions

In this paper, the structure-from-motion (SfM) photogrammetry technique is discussed and proposed as non-invasive and low-cost approach for three-dimensional reconstruction of pig body.

Preliminary tests carried out on a fiberglass reference model clearly show how SfM has the potential to provide a three-dimensional reconstruction with a consistent resolution, which can be applied for quantitative extraction of body parameters. However, application to live pigs poses limitations mainly ascribable to the movements (amplitude and frequency) of the animals themselves during images acquisitions. 

As a consequence, specific strategies have to be adopted, in order to minimize the effect of such movements on the reconstruction process. A possible approach can be based on the increase of the image acquisition frequency achievable, by way of example, through implementation of multiple cameras attached to a reference frame in the pig pen area.

Our future work will focus on automating the manual approach of estimations of images, in order to reduce the processing time required and facilitate the parameter extraction process. Additionally, further research will be done considering different pig breed-lines and pig barn environmental conditions.

## Figures and Tables

**Table 1:** Ages and weights of studied animals. For the fiberglass model, the weight of an animal with an equivalent size is reported.

Animal ID	Age (d)	Weight (kg)
Fiberglass reference	-	~209
1	1303	260
2	1537	268
3	1598	257
4	1497	272
5	501	234
6	724	256

**Table 2:** Comparison of performances of main 3D techniques, elaborated from the present study and from other works [34,35].

Technique	Instrumentation Costs ^1^ (EUR)	Resolution ^3^ (mm)	RMS Noise ^4^ (mm)	Scanning Time ^2^ (min)	Processing Time ^2^ (min)
Manual measurements	10–100	n.a.	n.a.	5–20	5–10
2D images	10–100	0.5 × 0.5 × n.a.	n.a.	3–10	10–40
Lidar	500–5000	1 × 1 × 3	2.5–6.0	3–30	30–90
Kinect v1	100–200	1 × 1 × 2	0.7–1.2	3–10	30–90
Structure from Motion	10–200	3 × 3 × 2	1.0–2.5	5–15	120–240

^1^ Includes tripod or frames; do not include computer and analysis software; ^2^ Includes only time to collect data or capture images; ^3^ Maximum achievable x-y-z resolutions with sensors at 1 m distance from the object; ^4^ Measured on a flat surface; n.a. Data calculation not possible and not available for that technique.

## References

[1] Wongsriworaphon A., Arnonkijpanich B., Pathumnakul S. (2015). An approach based on digital image analysis to estimate the live weights of pigs in farm environments. Comput. Electron. Agric..

[2] Bracke M.B., Metz J.H., Spruijt B.M., Schouten W.G. (2002). Decision support system for overall welfare assessment in pregnant sows B: validation by expert opinion. J. Anim. Sci..

[3] Nilsson M., Herlin A., Ardö H., Guzhva O., Åström K., Bergsten C. (2015). Development of automatic surveillance of animal behaviour and welfare using image analysis and machine learned segmentation technique. Animal.

[4] Stookey J.M., Gonyou H.W. (1994). The effects of regrouping on behavioral and production parameters in finishing swine. J. Anim. Sci..

[5] Menesatti P., Costa C., Antonucci F., Steri R., Pallottino F., Catillo G. (2014). A low-cost stereovision system to estimate size and weight of live sheep. Comput. Electron. Agric..

[6] Wang Y., Yang W., Winter P., Walker L. (2008). Walk-through weighing of pigs using machine vision and an artificial neural network. Biosyst. Eng..

[7] Apichottanakul A., Pathumnakul S., Piewthongngam K. (2012). The role of pig size prediction in supply chain planning. Biosyst. Eng..

[8] Wu J., Tillett R., McFarlane N., Ju X., Siebert J.P., Schofield P. (2004). Extracting the three-dimensional shape of live pigs using stereo photogrammetry. Comput. Electron. Agric..

[9] Lee J., Jin L., Park D., Chung Y. (2016). Automatic recognition of aggressive behaviour in pigs using a Kinect depth sensor. Sensors.

[10] Brandl N., Jorgensen E. (1996). Determination of live weight of pigs from dimensions measured using image analysis. Comput. Electron. Agric..

[11] Pope G., Moore M. (2002). DPI Pig Tech Notes: Estimating Sow Live Weights without Scales.

[12] Pezzuolo A., Guarino M., Sartori L., González L.A., Marinello F. (2018). On-barn pig weight estimation based on body measurement by means of a Kinect v1 sensor. Comput. Electron. Agric..

[13] Andújar D., Dorado J., Fernández-Quintanilla C., Ribeiro A. (2016). An approach to the use of depth cameras for weed volume estimation. Sensors.

[14] Dubbini M., Pezzuolo A., De Giglio M., Gattelli M., Curzio L., Covi D., Yezekyan T., Marinello F. (2017). Last generation instrument for agriculture multispectral data collection. CIGR J..

[15] Wang Z., Walsh K.B., Verma B. (2017). On-Tree Mango Fruit Size Estimation Using RGB-D Images. Sensors.

[16] Zhang H., Wei Q., Jiang Z. (2017). 3D Reconstruction of Space Objects from Multi-Views by a Visible Sensor. Sensors.

[17] Han J., Shao L., Xu D., Shotton J. (2013). Enhanced computer vision with Microsoft Kinect sensor: A review. IEEE Trans. Cybern..

[18] Kim J., Chung Y., Choi Y., Sa J., Kim H., Chung Y., Park D., Kim H. (2017). Depth-Based Detection of Standing-Pigs in Moving Noise Environments. Sensors.

[19] Kongsro J. (2014). Estimation of pig weight using a Microsoft Kinect prototype imaging system. Comput. Electron. Agric..

[20] Vranken E., Berckmans D. (2016). Precision livestock farming for pigs. Animal Front..

[21] Vázquez-Arellano M., Griepentrog H.W., Reiser D., Paraforos D.S. (2016). 3-D Imaging Systems for Agricultural Applications—A Review. Sensors.

[22] Jadlovský J., Jadlovská A., Jadlovská S., Čerkala J., Kopčík M., Čabala J., Vošček D. Research activities of the center of modern control techniques and industrial informatics. Proceedings of the 14th International Symposium on Applied Machine Intelligence and Informatics (SAMI).

[23] Sansoni G., Trebeschi M., Docchio F. (2009). State-of-the-art and applications of 3D imaging sensors in industry, cultural heritage, medicine, and criminal investigation. Sensors.

[24] Jay S., Rabatel G., Hadoux X., Moura D., Gorretta N. (2015). In-field crop row phenotyping from 3D modeling performed using Structure from Motion. Comput. Electron. Agric..

[25] Wróżyński R., Pyszny K., Sojka M., Przybyła C., Murat-Błażejewska S. (2017). Ground volume assessment using ’Structure from Motion’ photogrammetry with a smartphone and a compact camera. Open Geosci..

[26] Javernick L., Brasington J., Caruso B. (2014). Modelling the topography of shallow braided rivers using Structure-from-Motion photogrammetry. Geomorphology.

[27] Dai F., Lu M. (2010). Assessing the accuracy of applying photogrammetry to take geometric measurements on building products. J. Construct. Eng. Manag..

[28] Fathi H., Dai F., Lourakis M. (2015). Automated as-built 3D reconstruction of civil infrastructure using computer vision: Achievements, opportunities, and challenges. Adv. Eng. Inform..

[29] Brown D. (2010). Tracker Video Analysis and Modelling Tool.

[30] Savio E., De Chiffre L., Schmitt R. (2007). Metrology of freeform shaped parts. CIRP Ann. Manuf. Technol..

[31] Marchant J.A., Schofield C.P. (1993). Extending the snake image processing algorithm for outlining pigs in scenes. Comput. Electron. Agric..

[32] Schofield C.P., Marchant J.A., White R.P., Brandl N., Wilson M. (1999). Monitoring pig growth using a prototype imaging system. J. Agric. Eng. Res..

[33] Pastorelli G., Musella M., Zaninelli M., Tangorra F., Corino C. (2006). Static spatial requirements of growing-finishing and heavy pigs. Livest. Sci..

[34] Pezzuolo A., Guarino M., Sartori L., Marinello F. (2018). A Feasibility Study on the Use of a Structured Light Depth-Camera for Three-Dimensional Body Measurements of Dairy Cows in Free-Stall Barns. Sensors.

[35] Pezzuolo A., Giora D., Guo H., Ma Q., Guercini S., Sartori L., Marinello F. A comparison of low-cost techniques for three-dimensional animal body measurement in livestock buildings. Proceedings of the MetroAgriFor 2018: 1st Workshop on Metrology for Agriculture and Forestry.

[36] Westoby M.J., Brasington J., Glasser N.F., Hambrey M.J., Reynolds J.M. (2012). ‘Structure-from-Motion’ photogrammetry: A low-cost, effective tool for geoscience applications. Geomorphology.

[37] Shi C., Teng G., Li Z. (2016). An approach of pig weight estimation using binocular stereo system based on LabVIEW. Comput. Electron. Agric..

[38] Wang K., Guo H., Ma Q., Su W., Chen L., Zhu D. (2018). A portable and automatic Xtion-based measurement system for pig body size. Comput. Electron. Agric..

